# Phenotypic and genotypic resistance to bedaquiline in patients with multi-drug-resistant tuberculosis—experiences from Armenia

**DOI:** 10.1128/aac.01839-24

**Published:** 2025-04-09

**Authors:** E. Ardizzoni, W. Mulders, M. De Diego Fuertes, A. Hayrapetyan, A. Mirzoyan, J. Faqirzai, N. Khachatryan, I. Oganezova, F. Varaine, M. Bastard, P. Graulus, C. J. Meehan, L. Rigouts, B. C. de Jong, T. Decroo, C. Hewison, A. Van Rie

**Affiliations:** 1 Institute of Tropical Medicine, Antwerp, Belgium; 2 Médecins Sans Frontières 6440, Paris, France; 3 University of Antwerp Faculty of Medicine and Health Sciences 81844 , Wilrijk, Flanders, Belgium; 4 National Tuberculosis Control Centre of Armenia, Yerevan, Armenia; 5 National Reference Laboratory of Armenia, Yerevan, Armenia; 6 Médecins Sans Frontières 6440, Yerevan, Armenia; 7 Epicentre 55770, Paris, Île-de-France, France; 8 Nottingham Trent University Clifton Campus 124688, Nottingham, United Kingdom; 9 University of Antwerp 26660, Antwerp, Flanders, Belgium; Bill & Melinda Gates Medical Research Institute, Cambridge, Massachusetts, USA

**Keywords:** bedaquiline, mutations, WGS, MDR-TB, Armenia

## Abstract

Risk factors for baseline bedaquiline (BDQ) resistance, amplification during treatment, and correlations with treatment outcomes are not fully understood. This cohort included Armenian patients with multidrug-resistant TB predominantly fluoroquinolone-resistant enrolled between 2013 and 2015 in a BDQ compassionate use program. BDQ resistance at baseline and during treatment was assessed using MGIT (pDST_MGIT_), minimal inhibitory concentration in 7H11 (MIC_7H11_), and whole-genome sequencing. Risk factors, such as treatment effectiveness or stage of the disease, were analyzed for association with baseline BDQ resistance, acquired BDQ resistance, and treatment outcome. Among 39 patients, baseline BDQ resistance was 6% (2/33) by pDST_MGIT_ and 7% (2/29) by MIC_7H11_. All four baseline isolates with an *Rv0678* mutation were phenotypically resistant. During treatment, 48% of the patients acquired BDQ resistance by pDST_MGIT_, and 52% acquired mutations at various frequencies (97% in *Rv0678*). None of the factors significantly contributed to baseline or acquired BDQ resistance. Unfavorable treatment outcome (41%) was more frequent in the presence of acquired *Rv0678* mutations [odds ratio (OR) 132, 95% confidence interval (CI) 7.43, 2375], phenotypic BDQ resistance (OR 176, 95% CI 6.48, 2423), or MIC increase above or below the critical concentration (both OR 84.3, 95% CI 2.93, 2423) during treatment. For these highly treatment-experienced patients, low baseline prevalence but high incidence of acquired BDQ resistance was observed. Acquisition of mutations in BDQ candidate resistance genes, regardless of their frequency, or increased MICs during treatment, even below the critical concentration, should be seen as a warning sign of resistance amplification and increased risk of unfavorable treatment outcome.

## INTRODUCTION

Treating drug‐resistant tuberculosis is difficult. The use of bedaquiline (BDQ) results in improved treatment outcomes for treatment of multidrug-resistant (MDR-TB) (1) and is now recommended for all patients (2). Unfortunately, resistance has already become a concern, with the rate of acquired BDQ resistance being higher than that of acquired rifampicin resistance (3, 4). Currently, genes believed to be involved in BDQ resistance (BDQ candidate resistance genes) are *atpE*, *Rv0678* (*mmpR5*), *mmpS5*, *mmpL5*, *pepQ*, and *Rv1979*c (5). In clinical isolates, mutations are mainly found in the *Rv0678* gene, which encodes for a repressor of the mmpS5–mmpL5 efflux pump (6, 7). The association between BDQ genotype and phenotype is variable, ranging from a wild-type genome in phenotypically resistant isolates, mutations in *Rv0678* in isolates with a minimal inhibitory concentration (MIC) just below or above the critical concentration (CC) (8), and BDQ hyper-susceptibility when *Rv0678* mutations are present in combination with a variant in *mmpS5* (*Rv0677c*) or *mmpL5* (*Rv0676c*) (9). Further complicating the genotype–phenotype association is the observation that multiple *Rv0678* variants can be present in a single isolate, and that minority variants often appear and disappear in serial patient isolates (7, 10). In some studies, mutations that emerge and become fixed during treatment have been associated with unfavorable treatment outcomes (11, 12), while the significance of minority variants remains unclear.

Clofazimine (CFZ), an anti-leprosy drug (13) that is also used for treatment of RR-TB, shares the same efflux pump-mediated resistance mechanism with BDQ, resulting in cross-resistance between these two drugs (14–16). BDQ resistance has been reported in BDQ-naïve patients exposed to CFZ (7) and when CFZ is used together with BDQ (17), but the effect of exposure to CFZ on the emergence of BDQ resistance remains poorly quantified (6, 18).

In this study, we analyzed data from Armenia, where Médecins sans Frontières (MSF) in collaboration with the Ministry of Health and the National Tuberculosis Centre provided access to BDQ under compassionate use as part of a longer individualized regimen (19). We aimed to determine the baseline prevalence of mutations in BDQ candidate resistance genes, baseline prevalence of phenotypic BDQ resistance, and the association of baseline resistance with a history of CFZ exposure and treatment outcomes. We also aimed to describe changes during treatment, including acquisition of mutations in BDQ candidate resistance genes, acquisition of phenotypic BDQ resistance during treatment, increase of BDQ MIC below the CC (MIC creep), factors associated with these events, and their effect on treatment outcomes. Finally, we assessed the level of agreement between different phenotypic drug-susceptibility testing (pDST) and between phenotypic and genotypic methods and investigated partial growth on pDST_MGIT_ as an indication of heteroresistance and as a possible cause for observed discordances.

## MATERIALS AND METHODS

### Study population, setting, and data collection

Adults (≥18 years) who started treatment with BDQ under the compassionate use program in Armenia between May 2013 and April 2015 and who had at least one *Mycobacterium tuberculosis* (*Mtb*) isolate available for analysis at the Mycobacteriology Unit of the Institute of Tropical Medicine (ITM) in Antwerp, Belgium were eligible for inclusion in this retrospective analysis. Patients were eligible for the compassionate access program if they gave informed consent; their *Mtb* isolate was resistant to rifampicin, isoniazid, a fluoroquinolone (FQ) or second-line injectable (SLI) (i.e., kanamycin, amikacin, or capreomycin) or to both FQ and SLI; and a treatment regimen with BDQ, LZD, and at least one other likely effective drug could be designed.

Patient treatment characteristics, drug exposure, pDST results, and treatment outcomes were extracted from the on-site database. CFZ exposure was defined as ≥1 month of CFZ treatment before the start of the BDQ-containing regimen. For each patient, the number of effective drugs included in the treatment regimen was estimated. Drugs were considered effective if there was no evidence of resistance at baseline on pDST (on-site or at ITM) or by whole-genome sequencing (WGS). Effective drugs were classified as group A, B, or C according to the 2019 World Health Organization (WHO) classification (20). Capreomycin and kanamycin were excluded, as they were no longer considered effective for MDR/RR-TB treatment. Adherence to individual drugs was recorded as the proportion of prescribed doses taken, with ‘good adherence’ defined as an average adherence during the whole treatment of ≥80% (21). Using programmatic data sources, treatment outcomes were grouped as favorable (cure, treatment completion) or unfavorable (death due to any cause during treatment, lost to follow-up, treatment failure, which included culture conversion, followed by reversion).

An isolate was considered a baseline isolate when the sample was collected before or up to 7 days after the start of the BDQ-containing regimen. Results of cultures performed for treatment monitoring were used to determine time to culture conversion defined as the date of the first of two consecutive negative cultures (from samples collected at least 30 days apart) among patients with positive cultures at baseline (22). Culture reversion during treatment was defined as two consecutive positive cultures after culture conversion without evidence of re-infection defined by >12 single nucleotide polymorphism (SNP) difference on WGS. In the absence of WGS results, a single positive culture after conversion, followed by two or more negative cultures, was classified as most likely clerical error.

### Laboratory analyses

Available isolates were shipped from the National TB Reference Laboratory in Yerevan, Armenia to ITM for pDST for BDQ, MIC determination for BDQ and CFZ, and WGS analysis.

BDQ pDST was performed in the MGIT^960^ system (pDST_MGIT_) at a CC of 1.0 µg/mL (8). The exact growth units (GU) in the drug-containing tube were recorded (between 0 and 400). BDQ MIC was determined on 7H11 agar (MIC_7H11_) for concentrations ranging from 0.008 to 2.0 µg/mL, with a CC of 0.25 µg/mL. BDQ MIC_7H11_ and pDST_MGIT_ were repeated in case of discordance (i.e., when resistance was detected by pDST_MGIT_, but MIC_7H11_ was ≤0.125 µg/mL or when the isolate was susceptible by pDST_MGIT_, but MIC_7H11_ was ≥1.0 µg/mL). CFZ MIC was performed on 7H10 agar for concentrations ranging from 0.0313 to 2.0 µg/mL, with a CC of 1.0 µg/mL.

For WGS, isolates were sub-cultured on Löwenstein–Jensen medium, and colonies were transferred to 150 µL 0.5 M Tris–EDTA buffer. *Mtb* was lysed using a combination of enzymatic (lysozyme, RNase A, and proteinase K), heat (70°C for 5 min), and mechanical methods (FastPrep-24). *Mtb* DNA was extracted using the Maxwell 16 Cell DNA Purification Kit (Promega, Madison, USA). Following quality control, extracted gDNA was sequenced on an Illumina MiSeq instrument using the Illumina Nextera XT DNA Library Preparation Kit. Bioinformatics analysis was performed using the MAGMA pipeline (23) to detect both major (10–100%) and minor variant (<10%) allele frequency thresholds in genomic regions grouped as tier 1 and 2 genes according to their probability to contain resistance mutations, as classified by the WHO catalogue for mutations in *Mtb* Version 2 (5) or based on expert rules guided by literature review for cycloserine and para-aminosalicylic acid (PAS). No defined allelic frequency cutoffs are hard-coded in the MAGMA pipeline. Instead, a variant has to meet a series of criteria (mainly related to base calling quality, sequencing depth at the position of the variant) for the pipeline to report the variant in the drug resistance summary.

### Statistical analysis

The prevalence of mutations in BDQ candidate resistance genes at baseline was calculated as the proportion of patients whose baseline *Mtb* isolate contained one or more variants in BDQ candidate resistance genes among all patients with baseline WGS results available. Acquisition of mutations in BDQ candidate resistance genes on treatment was defined as the emergence of a new variant, regardless of its allele frequency or its presence in subsequent isolates. The acquisition of mutations in BDQ candidate resistance genes was calculated as the number of patients in whom ≥1 mutation not yet present in the baseline isolate was identified in ≥1 follow-up isolate (nominator) over the total number of patients with a WGS result on a follow-up isolate or for whom all follow-up isolates were negative for *Mtb* (denominator).

The prevalence of phenotypic BDQ resistance at baseline was calculated separately for pDST_MGIT_ and MIC_7H11_ as the proportion of patients with a phenotypically resistant isolate at baseline among all patients with baseline BDQ phenotypic results available. Acquisition of phenotypic resistance on pDST_MGIT_ was defined as a change from susceptible to resistant over time. An MIC_7H11_ increase was defined as an increase of ≥2 dilutions in BDQ-MIC_7H11_ and classified as either a rise from below to above the CC (acquisition of resistance) or an increase that remained below the CC (MIC creep) (24). The acquisition of phenotypic BDQ resistance on pDST_MGIT_ was defined as the number of patients in whom a change from susceptible to resistant pDST_MGIT_ result was observed during treatment (nominator) over the total number of patients with a pDST_MGIT_ result on a follow-up isolate or for whom all follow-up cultures were negative for *Mtb* (denominator). Similar calculations were applied to determine the incidence of acquisition of phenotypic resistance to BDQ on MIC7H11 and the incidence of BDQ MIC_7H11_ creep. Patients for whom baseline isolates were resistant by pDST_MGIT_ or showed an MIC above the CC were excluded from phenotypic resistance incidence estimates.

Logistic regression analysis was used to assess factors associated with the acquisition of mutations in BDQ candidate resistance genes, acquisition of phenotypic BDQ resistance (detected on pDST_MGIT_ or MIC_7H11_), and MIC creep. Covariates of interest were history of CFZ exposure, baseline CFZ resistance, inclusion of CFZ in the treatment regimen, number of effective drugs included in the regimen, smear positivity, presence of bilateral cavities, and adherence to BDQ, CFZ, and other drugs in the first 6 months of treatment. Factors investigated for association with unfavorable treatment outcome were phenotypic BDQ resistance at baseline, presence of mutations in BDQ candidate genes at baseline, and acquisition of mutations in BDQ candidate resistance genes or acquisition of phenotypic BDQ resistance during treatment. The associations were evaluated as crude odds ratios (OR) and their 95% confidence intervals (95% CI).

The agreement between phenotypic BDQ DST methods (MIC_7H11_ and pDST_MGIT_) and between phenotypic and genotypic methods was assessed by calculating Cohen’s kappa coefficients. Association between discordance and the exact growth unit on pDST_MGIT_ or presence of heteroresistance was assessed.

## RESULTS

### Patient characteristics and treatment

Of the 62 patients participating in the compassionate access program during the study period, 39 (62.9%) had at least one culture available and were included in the analysis. Almost all patients included in the analysis were male (95%) with an average age of 41 years; most were HIV-negative (95%) and had smear-positive (85%) or cavitary (100%) TB with bilateral (74%) lung damage (Table 1). All patients included in the study had a history of MDR-TB treatment with a regimen comprising FQ and SLI; 62% had been exposed to CFZ; and all were BDQ and LZD naive. Based on WGS results at baseline and DST results from previous treatment episodes, 95% (37/39) had resistance to at least rifampicin, isoniazid, and an FQ (Table S2). The 23 patients not included in the study had less severe diseases and were less likely to have prior CFZ exposure (Table S1).

**TABLE 1:** Patients' characteristics and previous and current treatment of 39 patients included in the analysis*^c^*^,*d*^

	*N* (%) or median (IQR)
Sociodemographic characteristics	
Age in years	41 (33–49)
Female	2 (5%)
Clinical characteristics	
Body mass index	19.2 (17.5–21.6)
Diabetes mellitus	3 (8%)
living with hiv (*n* = 38)^*a*^	2 (5%)
Hepatitis C serology positive	14 (36%)
TB treatment history	
Prior RR-TB treatment with FQ and SLI	39 (100%)
Prior RR-TB treatment with CFZ	24 (62%)
Current TB episode	
Cavitary TB	39 (100%)
Bilateral disease	29 (74%)
Smear microscopy positive	33 (85%)
Current TB episode: effective drugs (*n* = 35)^*b*^	
3 group A	1 (3%)
3 group A + 2 group B	1
2 group A	29 (74%)
2 group A + 2 group B + 1 group C	6
2 group A + 2 group B	5
2 group A + 1 group B + 1 group C	4
2 group A + 1 group B	12
2 group A + 1 group C	1
2 group A only	1
1 group A	5 (13%)
1 group A + 2 group B + 1 group C	1
1 group A + 1 group B + 2 group C	1
1 group A + 1 group B	1
1 group A only	2
Current TB episode: treatment outcome	
Favorable	23 (59%)
Cure	22
Treatment completed	1
Unfavorable	16 (41%)
Death	5
Treatment failure	10
Lost to follow-up	1

^*a*^ HIV status missing for one patient.

^*b*^ Listed for 35 patients with WGS data available.

^*c*^ CFZ = clofazimine; SLI = second-line injectables; FQ = fluoroquinolones; *N* = number; and IQR = interquartile range.

^*d*^ Drugs are classified based on WGS and phenotypic DST according to WHO drug groups A, B, and C.

### Treatment

All 39 patients initiated an individualized regimen containing BDQ and LZD combined with an additional two to six drugs selected based on prior drug exposure and DST results from prior treatment episodes. Cycloserine was used in 31 (80%) patients, CFZ in 30 (77%), PAS in 17 (44%), an SLI drug in 16 (41%), levofloxacin in 16 (41%), prothionamide in six (15%), and pyrazinamide in five (13%) patients. Patients received treatment for a median of 25 months (range 2 to 35), including a median of 6 months (range 2 to 16) of BDQ (Tables S3 and S4). The number of effective drugs included in the regimen could be assessed for 35 (90%) patients for whom a baseline isolate could be tested for MIC and WGS (Table S2). Overall, these patients received on average of four effective drugs (range 1 to 5). The inclusion of effective drugs by WHO class is shown in Table 1. Several patients received drugs that are no longer listed by the WHO as effective for treatment of TB but were recommended at the time.

### Treatment outcomes

Of the 39 patients, 23 (59.0%) had favorable treatment outcomes (22 were cured, and one completed treatment), and 16 (41.0%) had an unfavorable outcome (five died; 10 experienced treatment failure; and one was lost to follow-up) (Table 1; Tables S3 and S4).

Culture conversion occurred in 29/39 (74%) patients after a median of 3.5 months (QR 2.3–4.6) of treatment. Patients with a favorable outcome achieved culture conversion earlier (2.9 months; IQR 1.6–5.3) than those with an unfavorable treatment outcome (3.5 months, IQR 1.7–4.0). Among the 16 patients with unfavorable treatment outcome, nine had achieved culture conversion; for all, except one, culture reversion occurred at a median of 9.8 months [IQR 3.4–12.2] after conversion (Tables S3 and S4).

#### Association between baseline prevalence of mutations in BDQ candidate resistance genes, baseline phenotypic BDQ resistance, previous exposure to CFZ, and treatment outcomes

Of the 35 baseline isolates, four contained one or more mutations in *Rv0678*, corresponding to a 12% (95% CI 4.6–26.6) baseline prevalence of mutations in BDQ candidate resistance genes. All mutations occurred in the *Rv0678* gene: 137dupG (at 100% allele frequency), 198delG (93%) plus 130_133dupCTGG (4%), 139dupG (3%), and 269–272dupGCAC (2%) (Fig. S1). All four isolates were phenotypically resistant by either MIC_7H11_ or pDST_MGIT_, but none were resistant by both methods (Table 2). The baseline prevalence of phenotypic BDQ resistance was 2/35, 6% (95% CI 1.5–18.6) for either pDST_MGIT_ or MIC_7H11_ for a total prevalence of 4/35, 12% (4.6–26.6). The presence of genomic resistance to BDQ at baseline was observed both in isolates from patients with favorable and unfavorable treatment outcomes (OR 1.5, 95% CI 0.19, 12.2). The two patients whose isolate contained a fixed (≥90% allele frequency) variant had a favorable treatment outcome, while those with a minority variant had an unfavorable treatment outcome.

**TABLE 2:** Phenotypic resistance and presence of variants in BDQ candidate resistance genes at baseline and during treatment stratified by treatment outcome

				Treatment outcome		
		All patients *N*		Favorable	Unfavorable	OR (95% CI)
Baseline			Prevalence rate			
Mutation in *Rv0678^a^*^,*c*^ (WGS available for 34 patients)	Yes	4	11.7% (4.6–26.6)	2	2	1.5 (0.19, 12.2)
	No	30		18	12	
BDQ MIC_7H11_ ≥ 0.5 µg/mL (MIC available for 35 patients)	Yes	2	5.7% (1.5–18.6)	0	2	8.6 (0.38, 194)
	No	33		21	12	
BDQ DST_MGIT_ > 1.0 µg/mL (DST available for 31 patients)	Yes	2	6.4% (1.8–20.7)	2	0	0.28 (0.012, 6.35)
	No	29		17	12	
Follow-up			Incidence rate			
Acquisition of mutations (*n* = 25)^*b*^	Yes	13	52.0% (33.5–69.9)	1	12	132 (7.43, 2375)
	No^*b*^	12		11	1	
MIC_7H11_ increase ≥ 2 dilutions (*n* = 22)^*b*^	No increase^*b*^	12		11	1	84.3 (2.93, 2423) 84.3 (2.93, 2423)
	Increase above CC	5	22.7% (10.1–43.4)	0	5	
	Increase the below CC	5	22.7%, (0.1–43.4)	0	5	
Acquired resistance on DST_MGIT_ (*n* = 23)^*b*^	Yes	11	47.8% (29.2–67.0)	0	11	176 (6.48, 4797)
	No^*b*^	12		11	1	

^*a*^ No mutations detected in other BDQ candidate resistance-conferring genes.

^*b*^ Patients were classified as ‘not experiencing the event’ if there was no change in WGS, MIC, or MGIT results (second result in case of repetition) on any of the positive cultures or if all follow-up cultures were negative.

^*c*^ All four patients with mutations were phenotypically resistant by one of the two methods but none by both methods; BDQ = bedaquiline; MIC = minimal inhibitory concentration; MIC_7H11_ = MIC testing on 7H11 agar medium; CC = critical concentration; DST_MGIT_ = phenotypic drug-susceptibility testing by MGIT 960; N = number; OR = odds ratio; and CI = confidence interval.

Between the 35 patients with baseline isolates available, 23 had a history of CFZ exposure. Of these 23 baseline isolates, three contained an *Rv0678* mutation and were phenotypically resistant to both BDQ and CFZ. Among the 12 CFZ-naïve patients, one baseline isolate that contained a mutation in *Rv0678* was phenotypically resistant to BDQ but susceptible to CFZ. History of treatment with CFZ was not associated with genotypic or phenotypic BDQ resistance (OR 1.6; 95% CI 0.15–17.8).

#### Changes in BDQ phenotype or genotype on treatment, factors associated with these events, and their association with treatment outcomes

Of 66 follow-up culture isolates available, 63 isolates from 24 patients generated WGS results; 60 isolates from 24 patients had BDQ MIC_7H11_ results; and 55 isolates from 20 patients had BDQ pDST_MGIT_ results. The cumulative probabilities were 52% (95% CI 34–70; 13/25) for acquisition of mutations, 48% (95% CI, 29–67; 11/23) for acquisition of phenotypic resistance defined by pDST_MGIT_, 23% (95% CI, 10–43; 5/22) for acquisition of phenotypic resistance defined by MIC_7H11_, and 23% (95% CI, 10–43; 5/22) for MIC creep.

In the 63 isolates with WGS data, 35 (97%) new mutations in *Rv0678* (22 SNPs and 13 frameshifts) and one (3%) frameshift in *pepQ* emerged during treatment (Fig. S1; Table S5). The number of variants present ranged from one in 33 isolates (66%) to two in nine (18%) and more than two in eight isolates (16%) (Table S5). After acquisition, gain or loss of mutations over time was observed in 12 of the 13 patients with more than one follow-up isolate available. Mutations that replaced other mutations were generally present at higher allele frequency and conferred the same or higher MIC_7H11_ compared to the mutation lost. In other cases, low-allele frequency mutations gained in addition to fixed mutations were lost, or when mixed mutations amplified, only the ones with higher allele frequency tended to be maintained in subsequential isolates (examples in Fig. S2). Acquisition of mutations in candidate resistance genes for drugs other than BDQ was observed in 18 patients: CFZ (*n* = 11), cycloserine (*n* = 6, kanamycin (*n* = 2), LZD (*n* = 3), PAS (=1), and FQ (*n* = 1).

The distribution of baseline factors investigated was similar between patients whose follow-up Mtb isolate did or did not acquire mutations in BDQ candidate resistance genes, acquire phenotypic BDQ resistance, or MIC creep (Table 3).

**TABLE 3:** Risk factors for the acquisition of mutations in bedaquiline (BDQ) candidate resistance genes and BDQ minimal inhibitory concentration (MIC) increase during treatment^*a*^

		Acquisition of mutations in BDQ candidate resistance genes						Increase of BDQ MIC_7H11_						Acquisition of BDQ resistance in DST_MGIT_					
		Total	Yes		No		OR (95% CI)	Total	Increase		stable		OR (95% CI)	Total	BDQ S-R		BDQ S		OR (95% CI)
		N	N	%	N	%		N	N	%	N	%		N	N	%	N	%	
All patients		25	13	52	12	49		22	10	45	12	55		22	11	50	11	50	
History of CFZ treatment	Yes.	18	9	50	9	50	0.75 (0.13–4.36)	16	7	44	9	56	0.77 (0.11–5.10)	14	7	50	7	50	0.87 (0.15–4.87)
	no	7	4	57	3	43		6	3	50	3	50		8	4	50	4	50	
Baseline CFZ phenotypic resistance	yes	3	2	67	1	33	1.82 (0.14–23.3)	1	0	0	1	100	0.33 (0.01–9.15)	2	1	50	1	50	0.72 (0.03–13.45)
	no	21	11	52	10	48		20	10	50	10	50		19	10	53	9	47	
CFZ in regimen	yes	17	10	56	7	44	2.38 (0.42–13.4)	14	7	50	7	50	1.66 (0.28–9.82)	15	8	53	7	47	1.90 (0.32–11.00)
	no	8	3	38	5	62		8	3	38	5	62		7	3	438	4	57	
Number of effective drugs	<3	3	2	67	1	33	2.00 (0.15–25.40)	1	1	100	0	0	3.94 (0.14–108.09)	1	1	100	0	0	3.57 (0.12–97-23)
	≥3	22	11	50	11	50		21	9	43	12	57		21	10	48	11	52	
Smear positive	yes	18	11	61	7	39	3.14 (0.44–21.95)	15	8	53	7	47	2.28 (0.31–16.51)	16	9	56	7	44	1.68 (0.22–12.80)
	no	6	2	33	4	67		6	2	33	4	67		5	2	40	3	60	
Bilateral cavities	yes	17	11	65	6	35	5.50 (0.83–36.19)	16	9	56	7	44	13.00 (0.62–268.94)	18	10	56	8	44	5.00 (0.46–54-04)
	no	8	2	25	6	75		6	1	17	5	83		4	1	25	3	75	
Adherence to BDQ	<80%	4	4	100	0	0	13.59 (0.66–282.1)	4	4	100	0	0	14.53 (0.66–316.71	4	3	80	1	20	5.14 (0.46–56.89)
	≥80%	16	6	38	10	62		16	6	38	10	62		14	6	43	8	57	
Adherence to CFZ (month 0–6)	<80%	5	5	100	0	0	15.36 (0.69–338.50)	5	4	80	1	20	8.00 (0.59–106.94)	5	4	80	1	20	7.50 (0.62–90.65)
	≥80%	9	3	33	6	67		9	3	33	6	67		9	4	44	5	56	
Adherence to other drugs (month 0–6)	<80%	9	6	67	3	33	2.00 (0.35–11.36)	8	5	63	3	37	2.33 (0.37–14.61)	7	5	71	2	29	3.33 (0.47–23.47)
	≥80%	14	7	50	7	50		12	5	42	7	58		13	6	46	7	54	

^*a*^ CFZ = clofazimine; MIC = minimal inhibitory concentration; CFZ phenotypic resistance if MIC > 1.0 µg/mL; MIC_7H11_ = MIC testing on 7H11 agar medium; DST_MGIT_ = phenotypic drug-susceptibility testing by MGIT 960; *N* = number; OR = odds ratio; CI = confidence interval; adherence = percentage of prescribed doses taken; and month (0–6) = 6 months of treatment with BDQ.

Acquisition of mutations in *Rv0678* was mainly observed in follow-up isolates from patients with unfavorable treatment outcomes (OR 132.0, 95% CI 7.43, 2375): two of the 13 patients whose isolates acquired new mutations died, and 10 experienced treatment failure (Table 2). Acquisition of phenotypic BDQ resistance defined by pDST was also mainly observed in patients with unfavorable treatment outcomes: one of 11 patients acquiring phenotypic BDQ resistance died, and 10 experienced treatment failure (OR 176, 95% CI 6.48, 4797). Similarly, acquisition of phenotypic resistance defined by BDQ MIC_7H11_ and MIC_7H11_creep was more frequent in patients with unfavorable treatment outcomes, with all five patients who acquired phenotypic BDQ resistance experiencing an unfavorable outcome (one death and four treatment failures) (OR 84.3, 95% CI 2.93, 2423) and all patients who experienced BDQ MIC_7H11_ creep experiencing unfavorable treatment outcomes (two deaths, three treatment failures) (OR 84.3, 95% CI 2.93, 2423). All patients (11/11) with acquired pDST MGIT resistance had an unfavorable outcome.

#### Agreement between phenotypic tests (pDST_MGIT_ and MIC_7H11_) and between phenotypic and genotypic tests for assessment of BDQ resistance

**Fig 1:**
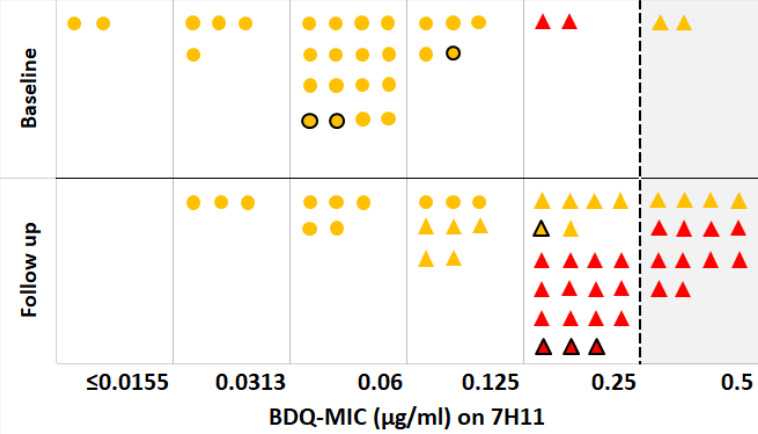
BDQ MIC on Middlebrook 7H11 agar for 82 MGIT BDQ-susceptible and -resistant isolates stratified by time of collection. Circle = wild-type isolate; triangle = isolate with mutations in BDQ candidate resistance genes; in yellow MGIT BDQ-susceptible and in red MGIT BDQ-resistant; black borders, results from test repetition; dotted line = critical concentration.

Of the 86 isolates with WGS results, paired MIC_7H11_ and pDST_MGIT_ results were available for 82 isolates (Fig. 1). Of these, phenotypic assays were repeated in seven because of discordant results (all resistant by pDST with MIC ≤ 0.125 µg/mL). Four of the seven repeated pDST_MGIT_ assays were susceptible, and three remained resistant. MIC was repeated for four isolates (one confirmed pDST susceptible and three resistant), increasing to 0.25 µg/mL. When taking these repeated results into account, 10 of 27 isolates BDQ resistant by pDST_MGIT_ were also resistant on MIC,_7H11_ and 49 of the 55 isolates susceptible by pDST _MGIT_ were also susceptible by MIC_7H11_, with a total of 23 (28%) isolates with discordant results between phenotypic methods. Agreement between the two phenotypic methods was only fair (*K* = 0.30). When considering changing the CC for MIC_7H11_ from the current WHO CC of 0.25 µg/mL to 0.125, the number of discordant results dropped to 12, and the level of agreement increased (*K* = 0.70). None of the WT strains would yield an MIC ≥ 0.25 µg/mL. The 12 remaining isolates with discordant results were susceptible on pDST_MGIT_ but resistant on MIC_7H11_ with MIC of 0.25 (*n* = 6) or 0.5 µg/mL (*n* = 6).

**Fig 2:**
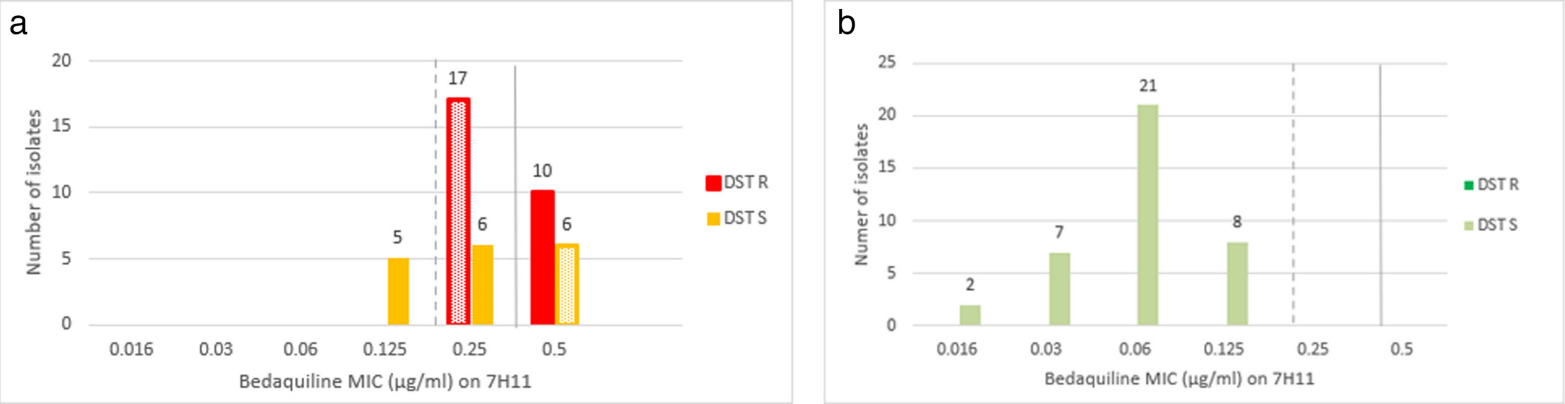
Results of BDQ MICs on Middlebrook 7H11 medium and phenotypic drug susceptibility tests on MGIT stratified by the presence of one or more mutations in a BDQ candidate resistance gene (a) (*n* = 44) and isolates with wild type for BDQ candidate resistance genes (b) (*n* = 38). Green: MGIT-susceptible results; full colored bars = concordant with MIC; dotted bars = discordant with MIC; full line = 0.25 µg/mL critical concentration; dotted line is a tentatively lowered critical concentration for 7h11 medium (to 0.125 µg/mL); DST-R = BDQ-resistant by MGIT-DST; DST-S = BDQ-susceptible by MGIT-DST.

Of the 44 isolates (Fig. 2a) with one or more mutations present in *Rv0678* (+/− *pepQ*), 27 (62%) were resistant on pDST _MGIT_, and 16 were resistant by MIC_7H11_ (using a CC of 0.25 µg/mL). All 38 wild-type isolates (Fig. 2b) were classified as susceptible on both pDST_MGIT_ and MIC_7H11_. This corresponded to a good agreement between WGS and pDST_MGIT_ (*K* = 0.60) and fair agreement between WGS and MIC_7H11_ (*K* = 0.35). Agreement between WGS and MIC_7H11_ increased to very good (*K* = 0.88) when the CC was lowered to 0.125 µg/mL.

Overall, of the 44 isolates with *Rv0678* (+/− *pepQ*) mutants, 17 (39%) were pDST_MGIT_ susceptible: 11 showed an MIC ≤ 0.25 µg/mL of which three showed GU at 84, 45, and 57. The other six isolates showed an MIC = 0.05 µg/mL, one with GU = 56. All 47 WT isolates showed MIC ≤ 0.25 µg/mL and GU = 0 in pDST_MGIT_.

## DISCUSSION

In this cohort of 39 highly treatment-experienced patients who received BDQ under compassionate use, 59% achieved a favorable treatment outcome similar to the 60% global treatment success rate for any RR-TB (25). Surprisingly, the baseline prevalence of mutations in BDQ candidate resistance genes was already 12% (95% CI 4.6–26.6), even though this was the first cohort to receive BDQ in Armenia. In this small cohort, exposure to CFZ was not more common in patients with baseline BDQ resistance, and treatment outcomes were similar for patients with or without baseline BDQ resistance. However, only in one case was BDQ resistance identified in a patient without prior CFZ exposure, as compared to three patients with BDQ resistance and prior exposure. While the interpretation remains uncertain, this observation highlights the potential impact of prior CFZ exposure on the selection of BDQ resistance. The rate of acquisition of BDQ resistance during treatment was high (52.0% for acquisition of mutations in *Rv0678*, 22.7% for acquisition of phenotypic resistance by MIC_7H11_, and 47.8% for acquisition of phenotypic resistance by pDST_MGIT_), and these events were more frequently observed in patients with unfavorable treatment outcomes. The cumulative probability of MIC creep was similar to the probability of MIC increase above the CC, and both events were more frequently observed in patients with unfavorable treatment outcomes. Suboptimal agreement between genomic and phenotypic DST methods and between different phenotypic DST methods was observed, with improvements in the level of agreement when the CC for MIC_7H11_ was decreased from 0.25 to 0.125 µg/mL, with no apparent loss of specificity.

Resistance to BDQ at baseline in BDQ-naive patients has been reported in several studies, albeit at lower frequencies, ranging from 3.8 to 8% (18, 26–29). In our cohort, all four isolates with a mutation present at baseline were phenotypically resistant by either pDST_MGIT_ or MIC_7H11_. Even if this observation may be an overestimate caused by selection bias and may be imprecise due to the limited number of isolates, this is in contrast to observations in prior studies where only a minority of baseline Rv0678 mutants showed elevated MIC (29). Two of the four patients with baseline resistance in our study had unfavorable treatment outcomes. While the presence of baseline mutations has been associated with worse treatment outcomes in some studies, even when conferring an MIC below the CC (30), some authors found comparable culture conversion rates in the presence of *Rv0678* mutations at baseline (11, 19). Future large studies should investigate if the presence of baseline variants in *Rv0678* is associated with unfavorable treatment outcomes and if this is dependent or not on the mutation type.

Acquisition of genotypic and/or phenotypic resistance to BDQ during BDQ- and LZD-containing treatment was higher in this cohort of treatment-experienced patients with FQ-resistant TB than what has been observed for FQ-resistant patients receiving the BPaL regimen (31). Except for one minor variant (5% allele frequency) in *pepQ* co-present with a mutation in *Rv0678*, all mutations acquired during treatment occurred in the *Rv0678* gene. Similar to findings from other studies, acquired mutations were often unfixed, occurring at allele frequency < 10% (7, 32). These minor variants did not always evolve toward a fixed variant and did not hinder the emergence of other mutations. Interestingly, when one mutation was replaced by another, the first mutant tended to have lower allele frequency and was associated with lower MIC compared to subsequent mutations. Similar to findings from other studies (7, 19), we observed that isolates with mutations acquired during treatment often resulted in an increased BDQ-MIC_7h11_ (below or above the CC), even in the presence of unfixed variants. The acquisition of mutations in candidate resistance genes for LZD was less common and never observed before the acquisition of BDQ resistance. One limitation of the study is that data do not reflect treatment with currently recommended regimens for MDR-TB. While our observation suggests that mutations causing BDQ resistance are acquired more frequently than mutations causing LZD resistance, the rate of mutation acquisition and the impact on treatment outcome may differ when new regimens that combine BDQ and LZD with pretomanid or delamanid are used.

Acquisition during treatment of genotypic or phenotypic resistance or MIC creep was strongly associated with unfavorable treatment outcomes. These results confirm findings from other studies, where up to 25% of patients with acquired BDQ resistance experienced unfavorable outcome (27). This highlights the need for early detection of acquired BDQ resistance. Analysis of larger data sets should help determine whether acquisition of *Rv0678* variants results in treatment failure. Future studies should also investigate if early diagnosis of acquisition of BDQ resistance and administration of individualized rescue regimens can result in better cure rates.

Accurate diagnosis of BDQ resistance, however, remains a challenge. We observed suboptimal agreement between phenotypic and genotypic DST. Phenotypic results for isolates with mutations in *Rv0678* were discordant between pDST_MGIT_ and MIC_7H11_ in 28.0% (23/82) isolates. Others also reported that MIC_7H11_ can misclassify isolates with *Rv0678* mutations as susceptible to BDQ mainly when the MIC is close to the CC (i.e., in the “area of technical uncertainty”) (7, 33). Agreement between pDST_MGIT_ and MIC_7H11_ was low but improved when the CC for 7H11 was lowered from 0.25 µg/mL, the interim CC recommended by WHO, to 0.125 µg/mL. Disagreement between phenotypic methods could be due to measurement at different time points, different cycles of subculturing prior to inoculation, and use of different media, which may have led to different rates of loss of specific mycobacterial sub-populations during subculturing (34).

Four isolates with *Rv0678* mutants showed limited growth in the drug-containing tube, below the 100 growth units (GU) cut-off for calling resistance, one with MIC above the CC. None of the WT isolates showed partial growth. While these results were interpreted as susceptible, the partial growth of mycobacteria may indicate an intermediate status of resistance. It may be more appropriate to report these results as borderline (35).

Several limitations of our study should be noted. First, the small number of patients included limits the robustness of our conclusions. Furthermore, there was a selection bias, with patients included in the study being culture-positive and having more severe disease compared to those not included. In addition, patients included in the study were also more likely to have had prior clofazimine exposure than those who were not included, which may have resulted in higher estimates of acquisition of resistance and unfavorable treatment outcomes. Third, as this was a secondary data analysis of samples and data collected for routine management of patients, baseline and follow-up isolates were not always available or stored. We, therefore, could not precisely establish the timing of acquisition of resistance, and the estimate of its cumulative probability may be biased.

In conclusion, patients with FQ-resistant MDR-TB should be assessed for the presence of BDQ resistance both at the time of starting a BDQ-containing regimen and when they fail to achieve timely culture conversion, as timely identification of BDQ resistance could contribute to improved treatment outcomes. An increase in MIC_7H11_, even if below the critical concentration, or acquisition of mutations in BDQ candidate resistance genes, regardless of their allele frequency, should be seen as a warning sign of resistance amplification and increased risk of unfavorable treatment outcome.

## SUPPLEMENTAL MATERIAL

Supplemental material aac.01839-24-s0001.docx Table S1 to S7; Figure S1 and S2.

ASM does not own the copyrights to Supplemental Material that may be linked to, or accessed through, an article. The authors have granted ASM a non-exclusive, world-wide license to publish the Supplemental Material files. Please contact the corresponding author directly for reuse.
