# Supplementary material for: Phenotypic and genotypic resistance to bedaquiline in patients with multi-drug-resistant tuberculosis—experiences from Armenia
Source: Antimicrob Agents Chemother. 2025 Apr 9;69(5):e01839-24. doi: 10.1128/aac.01839-24 (PMC12057352; doi:10.1128/aac.01839-24)
Supplement: Supplemental material — Table S1 to S7; Figure S1 and S2. [file aac.01839-24-s0001.docx]

**Table S1 Characteristics of patients included or excluded from the study**

| **Patient characteristics N (%)** | | |
| --- | --- | --- |
|  | **Included in the study** | **Not included** |
|  | **(N=39)** | **(N=23)** |
| Age, median (IQR) y | 41 (33-49) | 40 (29-52) |
| Female | 2 (5%) | 5 (22%) |
| Body mass index (BMI) | 19.2 (17.5-21.6) | 21.3 (19.3-23.9) |
| Diabetes mellitus | 3 (8%) | 2 (9%) |
| HIV positive | 2 (5%) | 2 (9%) |
| HIV data missing | 1(3%) | 0 |
| Hepatitis C serologically positive | 14 (36%) | 4 (18%) |
| Any cavities | 39 (100%) | 16 (70%) |
| Bilateral disease | 29 (74%) | 11 (48%) |
| Smear-positive | 33 (85%) | 6 (26%) |
| Drug exposure before BDQ-Rx | | |
| CFZ | 22 (76%) | 4 (17%) |
| SLI exposed | 39 (100%) | 23 (100%) |
| FQ exposed | 39 (100%) | 22 (96%) |
| DST profile at start of BDQ-Rx | | |
| MDR | 0 (0%) | 1 (4%) |
| MDR+INJ-R | 2 (5%) | 5 (22%) |
| MDR+FQ-R | 22 (56%) | 8 (35%) |
| MDR+INJ+FQ | 15 (39%) | 9 (39%) |

BDQ-Rx= Bedaquiline treatment; CFZ=clofazimine; SLI= second -line injectables; FQ=fluoroquinolones;

DST=drug susceptibility testing; MDR=multidrug resistance; N = number; IQR = interquartile range

**Table S2 Efficacy of drugs at baseline based on WGS, MIC and pDST results**

|  | BDQ | | | LZD | CFZ | | Cs | | LFX | | CM | | KM | AMK | | PAS | | Pto | PZA | | EMB | Individualized regimen | | | |
| --- | --- | --- | --- | --- | --- | --- | --- | --- | --- | --- | --- | --- | --- | --- | --- | --- | --- | --- | --- | --- | --- | --- | --- | --- | --- |
| ID | WGS | MIC | DST | WGS | WGS | DST | WGS | DST | WGS | DST* | WGS | DST | WGS | WGS | DST | WGS | DST | WGS | WGS | DST | WGS | Group A | Group B | Group C | other |
| L31 | wt | 0.03 | nd | wt | wt | nd | wt | nd | wt | S | - | - | - | - | - | MUT | nd | - | - | - | - | BDQ, LZD, LFX | CFZ, Cs | PAS |  |
| I23 | wt | 0.06 | S | wt | wt | 0.5 | wt | S | - | - | - | - | wt | - | - | MUT | S | - | - | - | - | BDQ, LZD | CFZ, Cs | PAS | KM |
| L09 | wt | 0.06 | S | wt | wt | 1.0 | wt | nd | MUT | R | - | - | MUT | - | - | wt | S | - | - | - | - | BDQ, LZD, LFX | CFZ, Cs | PAS | KM |
| L13 | wt | 0.015 | S | wt | wt | 1.0 | wt | nd | MUT | R | MUT | R | - | - | - | wt | S | - | - | - | - | BDQ, LZD, LFX | CFZ, Cs | PAS | CM |
| L27 | wt | 0.03 | S | wt | wt | 1.0 | wt | nd | - | - | MUT | R | - | - | - | wt | nd | - | - | - | - | BDQ, LZD | CFZ, Cs | PAS | CM |
| L10 | wt | 0.06 | S | wt | wt | 0.5 | wt | S | wt | R | - | - | - | - | - | wt | S | MUT | wt | R | - | BDQ, LZD, LFX | CFZ, Cs | PAS, Pto, PZA |  |
| I11 | wt | 0.06 | S | wt | wt | 1.0 | wt | S | MUT | R | - | - | - | - | - | - | - | MUT | - | - | - | BDQ, LZD, LFX | CFZ, Cs | Pto |  |
| I08 | wt | 0.06 | S | wt | wt | 1.0 | wt | nd | MUT | R | - | - | MUT | - | - | - | - | - | MUT | nd | - | BDQ, LZD, LFX | CFZ, Cs | PZA | KM |
| I13 | wt | 0.03 | S | wt | wt | 0.5 | wt | nd | MUT | R | - | - | MUT | - | - | - | - | - | - | - | - | BDQ, LZD, LFX | CFZ, Cs |  | KM |
| I27 | wt | 0.06 | S | wt | wt | 1.0 | wt | nd | - | - | wt | nd | - | - | - | - | - | - | - | - | - | BDQ, LZD | CFZ, Cs |  | CM |
| L21 | wt | 0.06 | S | wt | wt | 0.5 | wt | nd | - | - | wt | nd | - | - | - | - | - | - | - | - | - | BDQ, LZD | CFZ, Cs |  | CM |
| L12 | wt | 0.06 | S | wt | - | - | wt | S | - | - | - | - | wt | - | - | wt | R | - | wt | nd | wt | BDQ, LZD | Cs | PAS, PZA, EMB | KM |
| L16 | wt | 0.125 | S | wt | wt | 1.0 | MUT | nd | - | - | - | - | - | - | - | wt | nd | - | - | - | - | BDQ, LZD | CFZ, Cs | PAS |  |
| L06 | wt | 0.06 | S | wt | wt | 0.5 | - | - | - | - | wt | S | - | - | - | wt | S | - | - | - | - | BDQ, LZD | CFZ | PA*S* | CM |
| L24 | wt | 0.125 | S | wt | - | - | wt | S | - | - | wt | nd | - | - | - | wt | S | - | - | - | - | BDQ, LZD | Cs | PAS | CM |
| L07 | wt | 0.06 | S | wt | - | - | wt | nd | - | - | - | - | wt | - | - | wt | nd | - | - | - | - | BDQ, LZD | Cs | PAS | KM |
| I05 | wt | 0.06 | S | wt | wt | 0.5 | MUT | S | MUT | R | - | - | - | - | - | MUT | S | - | - | - | - | BDQ, LZD, LFX | CFZ, Cs | PAS |  |
| I25 | wt | 0.06 | S | nd | wt | nd | MUT | S | - | - | nd | S | - | - | - | - | - | - | MUT | nd | - | BDQ, LZD | CFZ, Cs | PZA | CM |
| I22 | wt | 0.015 | S | wt | wt | 2.0 | wt | S | - | - | - | - | - | - | - | MUT | R | - | - | - | - | BDQ, LZD | CFZ, Cs | PAS |  |
| I29 | wt | 0.125 | S | wt | wt | 2.0 | wt | nd | - | - | wt | S | - | - | - | - | - | - | - | - | - | BDQ, LZD | CFZ, Cs |  | CM |
| I33 | nd | 0.06 | S | wt | nd | 1.0 | - | - | - | - | - | - | wt | - | - | - | - | - | - | - | - | BDQ, LZD | CFZ |  | KM |
| I20 | wt | 0.06 | S | wt | wt | 0.5 | - | - | - | - | wt | nd | - | - | - | - | - | - | - | - | - | BDQ, LZD | CFZ |  | CM |
| I19 | wt | 0.125 | S | wt | wt | 1.0 | - | - | - | - | - | - | MUT | - | - | - | - | - | - | - | - | BDQ, LZD | CFZ |  | KM |
| I14 | wt | 0.03 | S | wt | wt | 1.0 | - | - | MUT | R | MUT | R | - | - | - | - | - | - | - | - | - | BDQ, LZD, LFX | CFZ |  | CM |
| L03 | wt | 0.06 | S | wt | wt | 1.0 | - | - | MUT | R | - | - | - | wt | R | - | - | - | - | - | - | BDQ, LZD, LFX | CFZ | AMK |  |
| L15 | wt | 0.06 | S | wt | - | - | wt | nd | MUT | R | - | - | wt | - | - | - | - | - | - | - | - | BDQ, LZD, LFX | Cs |  | KM |
| I07 | wt | 0.03 | S | wt | - | - | wt | S | MUT | R | - | - | MUT | - | - | - | - | - | - | - | - | BDQ, LZD, LFX | Cs |  | KM |
| L18 | wt | 0.125 | S | wt | - | - | wt | nd | - | - | wt | nd | - | - | - | MUT | nd | - | - | - | - | BDQ, LZD | Cs | PAS | CM |
| L11 | wt | 0.016 | nd | wt | - | - | - | - | - | - | - | - | wt | - | - | wt | S | - | - | - | - | BDQ, LZD |  | PAS | KM |
| I09 | wt | 0.03 | nd | wt | - | - | - | - | - | - | - | - | MUT | - | - | - | - | - | MUT | nd | - | BDQ, LZD |  | PZA | KM |
| L17 | wt | 0.03 | S | MUT | wt | 1.0 | wt | nd | - | - | wt | S | - | - | - | - | - | - | - | - | - | BDQ, LZD | CFZ, Cs |  | CM |
| I17 | MUT | 0.50 | S | wt | MUT | 1.0 | wt | nd | - | - | - | - | wt | - | - | - | - | wt | - | - | - | BDQ, LZD | CFZ, Cs | Pto | KM |
| I24 | MUT | 0.25 | R | wt | MUT | 2.0 | wt | S | - | - | - | - | - | - | - | MUT | R | - | - | - | - | BDQ, LZD | CFZ, Cs | PAS |  |
| I04 | MUT | 0.25 | R | wt | MUT | 2.0 | MUT | S | MUT | R | - | - | MUT | - | - | - | - | - | - | - | - | BDQ, LZD, LFX | CFZ, Cs |  | KM |
| L08 | MUT | 0.50 | S | wt | MUT | 2.0 | MUT | S | MUT | R | - | - | - | - | - | - | - | - | - | - | - | BDQ, LZD, LFX | CFZ, Cs |  |  |
| I12 | nd | 0.03 | nd | nd | nd | 1.0 | nd | nd | nd | R | nd | R | - | - | - | nd | nd | nd | - | - | - | BDQ, LZD, LFX | CFZ*,* Cs | PAS, Pto | CM |
| L22 | nd | nd | nd | nd | nd | nd | nd | nd | nd | S | - | - | nd | - | - | - | - | - | - | - | - | BDQ, LZD, LFX | CFZ, Cs |  | KM |
| I30 | nd | nd | nd | nd | nd | nd | nd | S | - | - | - | - | - | - | - | - | - | nd | - | - | - | BDQ, LZD | CFZ, Cs | Pto |  |
| L19 | nd | nd | nd | wt | - | - | nd | nd | - | - | nd | nd | - | - | - | - | - | nd | - | - | - | BDQ, LZD | Cs | Pto | CM |

WGS= whole genome sequencing; MIC= minimal inhibitory concentration; DST=drug susceptibility testing; S= susceptible; R= resistant; MUT= mutation; nd= not done; BDQ= bedaquiline; LZD=linezolid; CFZ= clofazimine; Cs=cycloserine; LFX=levofloxacin; CM= capreomycin; KM= kanamycin; AMK= amikacin; Pto= prothionamide; EMB=ethambutol; PZA=pyrazinamide; blue= susceptible ; red=resistant ; patients in gray= baseline WGS not available, number of effective drugs not established

**Table S3 Bedaquiline minimal inhibitory concentration values (µg/ml) and mutations in bedaquiline candidate resistance genes for *Mycobacterium tuberculosis* isolates from 23 patients with favorable outcome, by treatment phase.**

Patient IDs above the line indicate patients included in the analysis for amplification of phenotypic and genotypic resistance; colour cells: Red =culture negative at treatment start; orange=bedaquiline (BDQ)-based treatment; purple= treatment without BDQ ; white = treatment ended or no info; culture results: += positive but not further tested, -= negative, C=contaminated, empty cell= culture not performed; cells with black borders = month of culture conversion; NA = not available; WT = wild type sequence for BDQ candidate resistance genes; Mut (n) = mutation code, refer to Table S5.

**Table S4: Bedaquiline minimal inhibitory concentration values (µg/ml) and mutations in bedaquiline candidate resistance genes for *Mycobacterium tuberculosis* isolates from 16 patients with unfavorable outcome, by treatment phase.**

Patient IDs above the line indicate patients included in the analysis for amplification of phenotypic and genotypic resistance; colour cells: Red =culture negative at treatment start; orange=bedaquiline (BDQ)-based treatment; purple= treatment without BDQ ; white = treatment ended or no info; culture results: + = positive but not further tested, - = negative, C=contaminated, empty cell= culture not performed; cells with black borders = month of culture conversion LTFU= Lost to follow up; NA = not available; WT = wild type sequence for BDQ candidate resistance genes; Mut (n) = mutation code, refer to Table S5.

**Table S5 Overview of bedaquiline DST by MGIT^960^, minimal inhibitory concentration (MIC) on 7H11 agar and genotypic profile for BDQ candidate resistance genes from 34 baseline and 63 follow up isolates.**

| ***Mut***  **code** | **WGS (%)** | **N** |  | **MGIT DST (1µg/ml)** | | |  |  |  | | **Middlebrook 7H11 agar - MIC** | | | | | | |  | | |  | | **Concordance** | |
| --- | --- | --- | --- | --- | --- | --- | --- | --- | --- | --- | --- | --- | --- | --- | --- | --- | --- | --- | --- | --- | --- | --- | --- | --- |
|  |  |  |  | **S** | **R** | **NA** |  | **0.0008** | | **0.0155** | | **0.0313** | **0.06** | **0.125** | **0.25** | **0.5** | **1.0** | | **2.0** | **na** | |  | | **MGIT-MIC** |
| 1 | G24D (59) | 1 |  | 1 |  |  |  |  | |  | |  |  | 1 |  |  |  | |  |  | |  | | S |
| 2 | C46R (100) | 1 |  | 1 |  |  |  |  | |  | |  |  |  |  | 1 |  | |  |  | |  | | Discordant |
| 3 | S68G (99-100) | 2 |  |  | 2 |  |  |  | |  | |  |  |  | 2 |  |  | |  |  | |  | | Discordant |
| 4 | R82W (98-100) | 5 |  |  | 1 |  |  |  | |  | |  |  |  | 1 |  |  | |  |  | |  | | Discordant |
|  |  |  |  | 3 |  |  |  |  | |  | |  |  | 2 | 1 |  |  | |  |  | |  | |  |
|  |  |  |  |  |  | 1 |  |  | |  | |  |  |  |  |  |  | |  | 1 | |  | |  |
| 5 | V85F (100) | 1 |  |  | 1 |  |  |  | |  | |  |  |  |  | 1 |  | |  |  | |  | | R |
|  | R89L (86-100) | 6 |  |  | 4 |  |  |  | |  | |  |  |  | 3 | 1 |  | |  |  | |  | |  |
| 6 |  |  |  | 1 |  |  |  |  | |  | |  |  |  | 1 |  |  | |  |  | |  | | Discordant |
|  |  |  |  |  |  | 1 |  |  | |  | |  |  |  |  |  |  | |  | 1 | |  | |  |
| 7 | F100Y (100) | 1 |  |  | 1 |  |  |  | |  | |  |  |  |  | 1 |  | |  |  | |  | | R |
| 8 | Y157C (0.87)) | 1 |  | 1 |  |  |  |  | |  | |  |  |  | 1 |  |  | |  |  | |  | | S |
| 9 | 140dupA (100) | 4 |  |  | 4 |  |  |  | |  | |  |  |  | 3 |  |  | |  | 1 | |  | | Discordant |
| 10 | *137dupG (100)* | 1 |  |  | 1 |  |  |  | |  | |  |  |  | 1 |  |  | |  |  | |  | | Discordant |
| 11 | *139dupG (3)* | 2 |  | 2 |  |  |  |  | |  | |  |  |  | 1 | 1 |  | |  |  | |  | | Discordant |
| 12 | 144dupC (99-100) | 5 |  |  | 2 |  |  |  | |  | |  |  |  | 1 | 1 |  | |  |  | |  | | Discordant |
|  |  |  |  |  |  | 3 |  |  | |  | |  |  |  | 1 |  |  | |  | 2 | |  | |  |
| 13 | 198dupG (100) | 2 |  | 2 |  |  |  |  | |  | |  |  |  |  | 2 |  | |  |  | |  | | Discordant |
| 14 | *269_272dupGCAC (2)* | 1 |  | 1 |  |  |  |  | |  | |  |  |  |  | 1 |  | |  |  | |  | | Discordant |
| 15 | 19delG (28), E21K (48) | 1 |  |  | 1 |  |  |  | |  | |  |  |  | 1 |  |  | |  |  | |  | | Discordant |
| 16 | R89L(85) 139dupG (3),421_422dupGA (1) | 1 |  |  | 1 |  |  |  | |  | |  |  |  |  | 1 |  | |  |  | |  | | R |
| 17 | *198delG (93), 130_133dupCTGG (4)* | 1 |  |  | 1 |  |  |  | |  | |  |  |  | 1 |  |  | |  |  | |  | | Discordant |
| 18 | R135G (79), Q51* (27) | 1 |  | 1 |  |  |  |  | |  | |  |  | 1 |  |  |  | |  |  | |  | | S |
| 19 | L60P (43), 140dupA (58) | 1 |  |  | 1 |  |  |  | |  | |  |  |  |  | 1 |  | |  |  | |  | | R |
| 20 | R89L (85), 139dupG (12) | 1 |  |  | 1 |  |  |  | |  | |  |  |  | 1 |  |  | |  |  | |  | | Discordant |
| 21 | 19delG (67),198delG (9), 436(dupA (7) | 1 |  |  | 1 |  |  |  | |  | |  |  |  |  | 1 |  | |  |  | |  | | R |
| 22 | L154P (51), 144dupC (35), 198delG (11) | 1 |  |  | 1 |  |  |  | |  | |  |  |  |  | 1 |  | |  |  | |  | | R |
| 23 | L60R (11), Y92 (13), 141_142 insTC (20),198 delG (17), A102V(7), R94W (9), L154P (10), 274_287dup14bp(2) | 1 |  |  | 1 |  |  |  | |  | |  |  |  |  | 1 |  | |  |  | |  | | R |
| 24 | 141_142 dupTC (11), R94W (16), 144dupC (37), insC424 (11), 321dupC (3) | 1 |  |  | 1 |  |  |  | |  | |  |  |  |  | 1 |  | |  |  | |  | | R |
| 25 | V20F (26), 19delG (59), S52F (2), 198dupG (5), 435dupT(2) | 1 |  |  | 1 |  |  |  | |  | |  |  |  | 1 |  |  | |  |  | |  | | Discordant |
| 26 | G25S (49), 198delG (30), G87W (11), 137dupG (2), S53L (5) | 1 |  | 1 |  |  |  |  | |  | |  |  |  | 1 |  |  | |  |  | |  | | S |
| 27 | G25S(47), 198delG (10) | 1 |  | 1 |  |  |  |  | |  | |  |  |  | 1 |  |  | |  |  | |  | | S |
| 28 | N70D (83-95),435dupT (0.09) | 2 |  | 1 |  |  |  |  | |  | |  |  | 1 |  |  |  | |  |  | |  | | Discordant |
|  |  |  |  |  | 1 |  |  |  | |  | |  |  |  | 1 |  |  | |  |  | |  | |  |
| 29 | N70D (0.95),Glu49* (0.05) | 1 |  | 1 |  |  |  |  | |  | |  |  |  |  | 1 |  | |  |  | |  | | Discordant |
| 30 | R82W (100), pepQ 964delG (5) | 1 |  |  | 1 |  |  |  | |  | |  |  |  | 1 |  |  | |  |  | |  | | Discordant |
|  | WT^£^ | 47 |  | 38 |  |  |  |  | | 2 | | 7 | 21 | 8 |  |  |  | |  |  | |  | | S |
|  |  |  |  |  |  | 9 |  |  | | 2 | | 3 | 2 | 1 |  |  |  | |  | 1 | |  | |  |

Nr = number of isolates; MGIT^960^ = automated DST in Mycobacteria Growth Indicator Tube; R = resistant; S = susceptible; Mut = mutation; WGS = whole genome sequencing (if not specified, the mutation occurred in Rv0678); Between brackets, frequency as percentage, or their range for mutations found in multiple isolates; NA = not applicable because pDST not available; in italic, mutations in baseline isolates; ^£^= 30 are baseline isolates

**Table S6 Risk factors for *Rv0678*-mutation amplification and BDQ-MIC increase in 23 patients with favorable outcome**

| **ID** | **Cfz previous exposure** | **Baseline Cfz MIC**  **(µg/ml)** | **Cfz in MDR Rx** | **N effective drugs (class A,B,C) ^1^** | **Smear at start of BDQ Rx** | **Bilateral cavities** | **DOT (%)** | | | **BDQ genotype** | **BDQ phenotype** | | **Outcome** |
| --- | --- | --- | --- | --- | --- | --- | --- | --- | --- | --- | --- | --- | --- |
|  |  |  |  |  |  |  | **Mean BDQ 6M** | **Mean CFZ 6 M** | **Mean 6M Rx except BDQ** |  | **MIC** | **MGIT DST** |  |
| I33 | no | 1 | yes | 3 | neg | No | 92 | 94 | 92 | stable | stable | S | cured |
| L12 | yes | 0.5 | no | 5 | neg | Yes | na | na | na | stable | stable | S | cured |
| L17 | yes | 1 | yes | 3 | scanty | Yes | 100 | 95 | 94 | stable | stable | S | cured |
| I29 | yes | 2 | yes | 3 | 3+ | No | na | na | na | stable | stable | S | cured |
| L07 | yes | 0.5 | no | 4 | 2+ | Yes | 86 | na | 80 | stable | stable | S | cured |
| L18 | no | 0.5 | no | 3 | 3+ | Yes | 88 | na | 77 | stable | stable | S | cured |
| I11 | yes | 1 | yes | 4 | 3+ | No | 93 | 92 | 85 | stable | stable | S | cured |
| L13 | yes | 1 | yes | 5 | neg | Yes | 95 | 97 | 93 | stable | stable | S | cured |
| I27 | no | 1 | yes | 4 | na | Yes | 100 | 100 | 86 | stable | stable | S | cured |
| I12 | yes | 1 | yes | 6 | neg | No | 84 | na | 77 | stable | stable | na | completed |
| L11 | yes | na | no | 3 | 3+ | No | 97 | 98 | 90 | stable | stable | na | cured |
| I24^$^ | yes | >2 | yes | 2 | 3+ | No | 92 | 90 | 80 | amplified | na | na | cured |
| L19 | no | na | no | 5 | 2+ | Yes | 92 | na | 86 | na | na | S | cured |
| I30 | no | na | yes | 5 | 2+ | Yes | 99 | 97 | 95 | na | na | na | cured |
| L31 | no | na | yes | 5 | 1+ | Yes | 97 | 95 | 86 | na | na | na | cured |
| I22 | no | 2 | yes | 3 | 3+ | Yes | 100 | 100 | 98 | na | na | na | cured |
| I23 | no | 0.5 | yes | 5 | 1+ | No | 100 | 100 | 100 | na | na | na | cured |
| L21 | yes | 0.5 | yes | 4 | 3+ | Yes | 100 | 99 | 98 | na | na | na | cured |
| I19 | yes | 1 | yes | 3 | 1+ | Yes | 96 | 96 | 93 | na | na | na | cured |
| I05 | yes | 0.5 | yes | 3 | 3+ | Yes | 95 | 83 | 83 | na | na | na | cured |
| L16 | yes | 1 | yes | 4 | scanty | Yes | 100 | 100 | 94 | na | na | na | cured |
| I14 | yes | 1 | yes | 3 | 1+ | No | 98 | 95 | 95 | na | na | na | cured |
| I04^$^ | yes | >2 | yes | 1 | 1+ | Yes | 61 | 65 | 60 | na | na | na | cured |

**Table S7 Risk factors for *Rv0678*-mut amplification and BDQ-MIC increase in 16 patients with unfavorable outcome**

| **ID** | **Cfz previous exposure** | **Baseline Cfz MIC** | **Cfz in MDR Rx** | **N effective drugs (class A,B,C) ^1^** | **Smear at BDQ Rx start** | **Bilateral cavities** | **DOT (%)** | | | **BDQ genotype** | **BDQ phenotype** | | **Outcome** |
| --- | --- | --- | --- | --- | --- | --- | --- | --- | --- | --- | --- | --- | --- |
|  |  |  |  |  |  |  | **mean BDQ 6M** | **mean CFZ 6 M** | **mean Rx 6M except BDQ** |  | **MIC** | **MGIT DST** |  |
| I09 | yes | 1 | no | 2 | scanty | No | 81 | na | 41 | stable | moderate | na | died |
| L24 | yes | 0.5 | no | 4 | 3+ | Yes | 95 | 67 | 79 | amplified | stable | S-R | failed |
| L09 | yes | 1 | yes | 5 | 2+ | yes | 92 | 94 | 92 | amplified | increased | S-R | failed |
| L06 | yes | 0.5 | yes | 4 | 3+ | Yes | 74 | 70 | 66 | amplified | moderate | S | died |
| L15 | yes | 0.5 | no | 3 | 2+ | Yes | 97 | na | 94 | amplified | increased | S-R | failed |
| I07 | yes | 0.5^&^ | no | 3 | neg | Yes | 83 | na | 84 | amplified | increased | S-R | failed |
| L10 | yes | 0.5 | yes | 5 | neg | Yes | 74 | 74 | 70 | amplified | moderate | S-R | failed |
| L03 | no | 1 | yes | 3 | 3+ | Yes | 75 | 69 | 72 | amplified | increased | S-R | failed |
| I20 | no | 0.5 | yes | 3 | 3+ | Yes | 89 | 88 | 89 | amplified | moderate | S-R | failed |
| I13 | no | 0.5 | yes | 4 | 2+ | Yes | 75 | 73 | 69 | amplified | increased | S-R | died |
| L27 | yes | 1 | yes | 5 | 3+ | Yes | 96 | 93 | 80 | amplified | moderate | S-R | failed |
| L08*^$^ | yes | 2 | yes | 1 | 2+ | Yes | 72 | 75 | 74 | amplified | na | S-R | failed |
| I17*^$^ | no | 0.5 | yes | 4 | 1+ | No | 87 | 97 | 95 | amplified | na | S-R | failed |
| I08 | no | 1 | yes | 4 | 1+ | Yes | 98 | 93 | 90 | na | na | na | LTFU |
| L22 | no | na | yes | 6 | 1+ | Yes | 72 | 14 | 64 | na | na | na | died |
| I25 | no | 2 | yes | 3 | 3+ | Yes | 90 | 88 | 89 | na | na | na | died |

Patients with baseline isolate and negative cultures at follow up were assumed to be stable for mutation amplification and BDQ-MIC increase. For patients underlined, baseline isolate was not available.

$=Patents with baseline mutations in BDQ candidate resistance genes; *=Patients with baseline BDQ-MIC at 0.5µg/ml ; & baseline isolate not available, MIC result from the first follow up

1 Based on phenotypic/genotypic results at ITM and on site (Table S2)

N = number; BDQ = bedaquiline; Cfz = clofazimine; CFZ bold= resistant; smear in ()= sample collected after BDQ Rx start;

DST = drug-susceptibility; Rx = treatment; FQ-R = multidrug-resistant and resistant to fluoroquinolone (FQ: ofloxacin, moxifloxacin or LFX); SLI-R = multidrug-resistant and resistant to second-line injectable drugs (SLI: KAN or AMK); MDR = multidrug resistant and resistant to FQ and SLI

S=susceptible at baseline and culture negative or, if positive still susceptible; S-R= baseline susceptible but R at least for one follow up

LTFU = lost to follow up; DOT= amount of prescribed drug taken by the patient, expressed as percentage

**Figure S1 Mutations in the *Rv0678* gene**


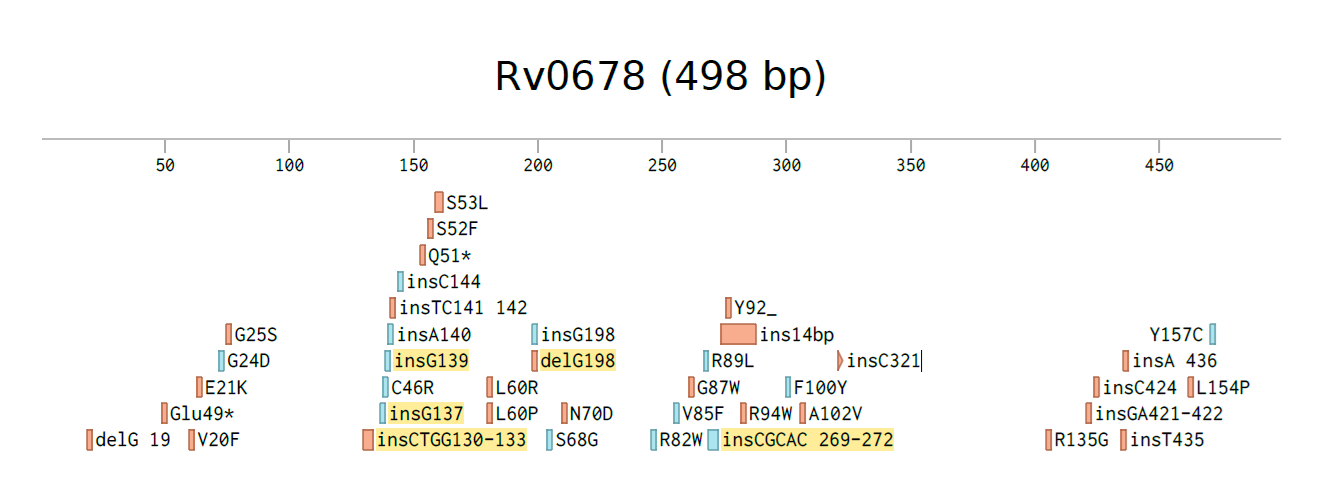


Orange: mutations found only in combination, blue: mutations found at least once alone. Highlighted in yellow: mutations found at baseline **Figure S2 Frequency of transient and minority *Rv0678* mutations versus**

**bedaquiline susceptibility**

Patient with replacing *Rv0678* mutations during treatment

Patient with *Rv0678* mutation maintained during treatment, while another

mutation appeared transiently

Patient with *Rv0678*-mut amplified as mixed

X-axis depicts only phases at which isolates were available. B= baseline; F1=BDQ-Rx phase;

F2= MDR-Rx phase; F3=Post MDR-Rx phase; F4= New BDQ-Rx phase. In brackets after the phase

is indicated the month of treatment. When no *Rv0678*-mut is reported, the isolate was WT.

MIC= minimal inhibitory concentration; pink circle= isolate carrying minority variants i.e. *Rv0678*-mut

detected at a frequency between 5% and 10%
